# Giant coronary artery aneurysm occluded completely by a thrombus

**DOI:** 10.1093/jscr/rjae355

**Published:** 2024-05-30

**Authors:** Shinichi Ishida, Genki Maeno, Aoi Kato, Yuson Wada, Hideyuki Okawa, Takahisa Sakurai, Toshimichi Nonaka

**Affiliations:** Department of Cardiovascular Surgery, JCHO Chukyo Hospital, 1-1-10 Sanjo, Minamiku, Nagoya, Aichi 457-8510, Japan; Department of Cardiovascular Surgery, JCHO Chukyo Hospital, 1-1-10 Sanjo, Minamiku, Nagoya, Aichi 457-8510, Japan; Department of Cardiovascular Surgery, JCHO Chukyo Hospital, 1-1-10 Sanjo, Minamiku, Nagoya, Aichi 457-8510, Japan; Department of Cardiovascular Surgery, JCHO Chukyo Hospital, 1-1-10 Sanjo, Minamiku, Nagoya, Aichi 457-8510, Japan; Department of Cardiovascular Surgery, JCHO Chukyo Hospital, 1-1-10 Sanjo, Minamiku, Nagoya, Aichi 457-8510, Japan; Department of Cardiovascular Surgery, JCHO Chukyo Hospital, 1-1-10 Sanjo, Minamiku, Nagoya, Aichi 457-8510, Japan; Department of Cardiovascular Surgery, JCHO Chukyo Hospital, 1-1-10 Sanjo, Minamiku, Nagoya, Aichi 457-8510, Japan

**Keywords:** coronary artery aneurysm, coronary artery embolism, coronary artery bypass grafting, coronary aneurysm resection

## Abstract

A coronary artery aneurysm is an uncommon vascular disorder, and it can be a life-threatening disease when associated with rupture or an embolism. A 52-year-old man was found to have a 50-mm coronary artery aneurysm at the right coronary artery, and the aneurysm was completely occluded by a thrombus. He had no symptoms after arriving at our hospital, and his hemodynamics was stable. Therefore, initially, we administered anticoagulation therapy involving heparin. After therapy, the distal coronary artery was detected when the thrombus dissolved, and elective surgery was planned. Coronary artery bypass grafting, ligation of the inflow and outflow vessels, and resection of the aneurysm were performed. Early anticoagulation therapy and surgical aneurysm resection were effective for treating the completely occluded coronary artery aneurysm. We herein report this rare case of a giant coronary artery aneurysm occluded completely by a thrombus and treated successfully by anticoagulation therapy and surgical aneurysm resection.

## Introduction

A coronary artery aneurysm (CAA) is an uncommon vascular disorder, and it can be a life-threatening disease when associated with rupture or an embolism [1]. In particular, a giant CAA that >20 mm is very rare. Moreover, a CAA completely occluded by a thrombus is even more uncommon. We herein present a rare case of a patient with a 50-mm CAA at the right coronary artery (RCA), which was completely occluded by a thrombus at detection.

**Figure 1:**
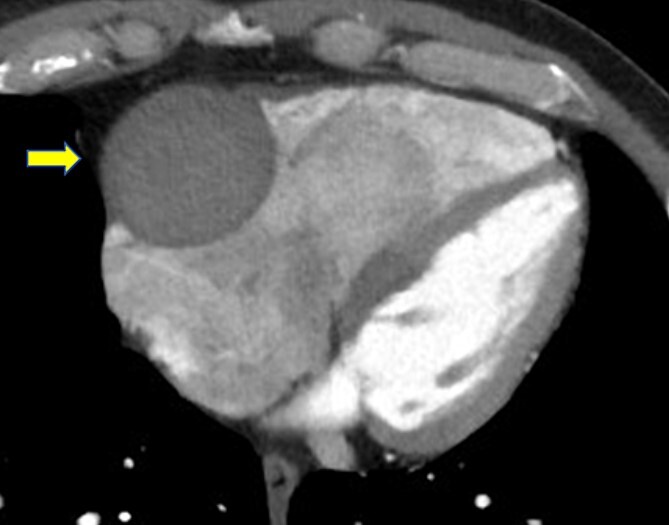
Enhanced computed tomography shows a giant coronary artery aneurysm (arrow) at the right coronary artery, which is occluded completely by a thrombus.

**Figure 2:**
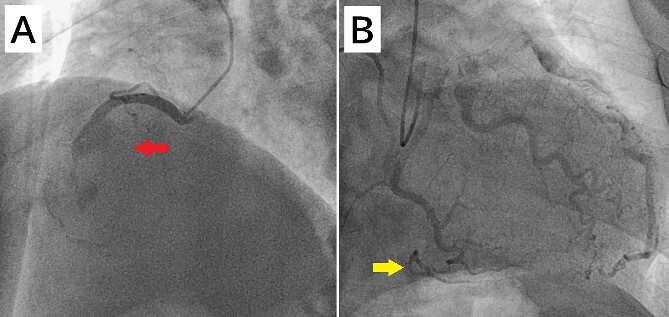
(A) Coronary angiography shows occlusion of the right coronary artery proximal to the aneurysm (arrow). (B) The artery distal to the aneurysm shows contrast via a collateral artery from the left circumflex artery (arrow).

**Figure 3:**
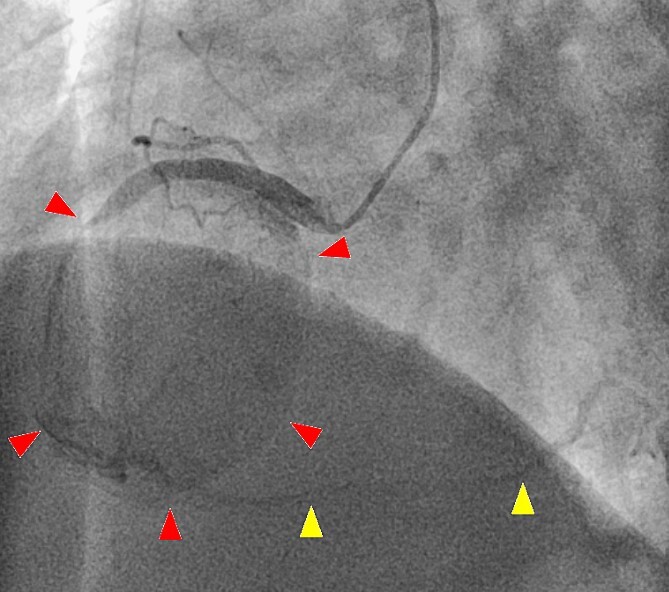
A second coronary artery angiography shows slight contrast in the aneurysm (arrow head) and in the distal coronary artery (arrow head).

Case report:

A 52-year-old man with transient ischemic attack was transferred to our hospital. He had a history of hypertension and dyslipidemia. He was conscious and had no particular symptoms after arriving at our hospital; however, an electrocardiogram showed ST-segment elevation in leads II, III, aVF, and V1–4. Enhanced computed tomography revealed a giant CAA at the RCA (Fig. 1). The aneurysm measured 50 mm in diameter and was completely occluded by a thrombus. Additionally, the coronary artery distal from the CAA did not show contrast. Emergency coronary angiography was performed. The RCA was occluded at segment #2 proximal to the CAA, and the CAA did not show contrast (Fig. 2A); however, the artery distal to the CAA showed contrast via a collateral artery from the left circumflex artery (Fig. 2B). Anticoagulation therapy involving intravenous heparin was started. After several hours, the ST-segment elevation disappeared quickly, and there were no particular symptoms. The creatine kinase level spiked to a maximum of 1475 IU/L, which then decreased to 432 IU/L on the next day. Four days after starting therapy, enhanced computed tomography and coronary angiography were performed again. They showed slight contrast in the CAA and the distal coronary artery (Fig. 3). Thus, surgery was performed to prevent the CAA from rupturing.

**Figure 4:**
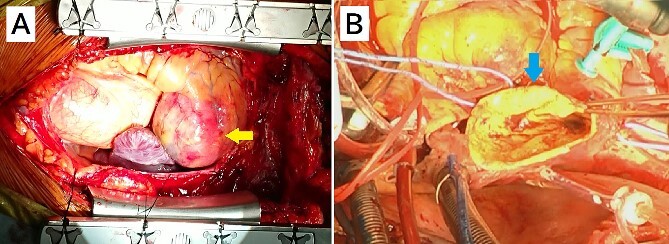
Intraoperative image from the surgeon’s perspective. (A) There is a giant coronary artery aneurysm (arrow) on the right ventricle. (B) A view of the inside of the aneurysmal sac (arrow).

After a median sternotomy, the right gastroepiploic artery was harvested. Cardiopulmonary bypass was established with ascending aortic cannulation and bicaval drainage. The giant CAA was present at the right ventricle (Fig. 4A). The inflow and outflow coronary arteries surrounding the CAA were identified, and the heart was arrested with antegrade cardioplegia. Coronary artery bypass grafting (CABG) was performed (right gastroepiploic artery–RCA distal to the CAA), and the CAA was cut after ligating the inflow and outflow vessels. The inner area of the CAA was filled with a thrombus, and the thrombus was removed immediately. There was no ostium of the other vessels (Fig. 4B); thus, the CAA wall was resected and the remnant wall was sutured. The postoperative course was uneventful. Postoperative computed tomography showed shrinkage of the CAA and no contrast in it, and confirmed patency of the bypass graft. The histopathological findings of aneurysm wall revealed that it was the pseudoaneurysm.

## Discussion

A CAA can be induced by atherosclerosis, Kawasaki disease, trauma, infection, etc. [2]. In the present case, the patient had hypertension and dyslipidemia, with no other relevant medical history. Therefore, the patient’s CAA was assumed to be associated with atherosclerosis.

The treatment strategy for a CAA is controversial, and several approaches have been reported, including conservative treatment with anticoagulation, catheter intervention or surgery, and emergent or elective intervention [3, 4]. Most reports have mentioned non-occluded or partially occluded CAAs. However, in the present case, the CAA was occluded completely by a thrombus when it was detected. Our patient had no symptoms after arriving at our hospital, and his hemodynamics was stable. In addition, the coronary artery distal from the aneurysm was unclear, and it was probably small. Therefore, we first decided to perform anticoagulation therapy. As a result, the distal coronary artery was detected when the thrombus dissolved, and elective surgery could be planned. There is a paucity of data and no randomized controlled study available to determine their efficacy, specifically in coronary aneurysm patients. In this case, it showed that early anti-coagulants therapy would be effective in patients with large coronary aneurysm occluded by a thrombus. We assumed that the occlusion of RCA has less influence for blood supply to anterior and lateral left ventricle, which was evident from the electrocardiogram showing ST-elevation in, leads V1-V4. Regarding anticoagulation therapy, aspirin, warfarin, and heparin are common pharmacological treatments to dissolve a thrombus and promote ischemia reperfusion in cases of a coronary aneurysm. We used intravenous heparin early, and it was effective against the thrombus.

Regarding the surgical strategy for a CAA, most reports describe ligating the CAA or inflow and outflow vessels at the same time as CABG, closing the CAA with a stent through catheter intervention, performing CABG followed by stenting, etc. [4–6]. In the present case, the CAA was completely occluded by a thrombus, and the presence of inflow vessels other than the main coronary artery was not clear. Therefore, to surely stop blood flow into the CAA, we opened the lumen of the CAA and checked that there was no inflow vessel. With this approach, we could close the ostium of other inflow vessels easily if present and perform CABG if necessary.

In conclusion, in patients with a CAA occluded by a thrombus, early anticoagulation therapy is effective. Furthermore, to surely stop blood flow into the CAA, it is important to open it and check that there is no other inflow vessel.
